# Evaluating Web-Based Automatic Transcription for Alzheimer Speech Data: Transcript Comparison and Machine Learning Analysis

**DOI:** 10.2196/33460

**Published:** 2022-09-21

**Authors:** Thomas Soroski, Thiago da Cunha Vasco, Sally Newton-Mason, Saffrin Granby, Caitlin Lewis, Anuj Harisinghani, Matteo Rizzo, Cristina Conati, Gabriel Murray, Giuseppe Carenini, Thalia S Field, Hyeju Jang

**Affiliations:** 1 Vancouver Stroke Program and Division of Neurology Faculty of Medicine University of British Columbia Vancouver, BC Canada; 2 Department of Computer Science Faculty of Science University of British Columbia Vancouver, BC Canada; 3 School of Computing University of the Fraser Valley Abbotsford, BC Canada

**Keywords:** Alzheimer disease, mild cognitive impairment, speech, natural language processing, speech recognition software, machine learning, neurodegenerative disease, transcription software, memory

## Abstract

**Background:**

Speech data for medical research can be collected noninvasively and in large volumes. Speech analysis has shown promise in diagnosing neurodegenerative disease. To effectively leverage speech data, transcription is important, as there is valuable information contained in lexical content. Manual transcription, while highly accurate, limits the potential scalability and cost savings associated with language-based screening.

**Objective:**

To better understand the use of automatic transcription for classification of neurodegenerative disease, namely, Alzheimer disease (AD), mild cognitive impairment (MCI), or subjective memory complaints (SMC) versus healthy controls, we compared automatically generated transcripts against transcripts that went through manual correction.

**Methods:**

We recruited individuals from a memory clinic (“patients”) with a diagnosis of mild-to-moderate AD, (n=44, 30%), MCI (n=20, 13%), SMC (n=8, 5%), as well as healthy controls (n=77, 52%) living in the community. Participants were asked to describe a standardized picture, read a paragraph, and recall a pleasant life experience. We compared transcripts generated using Google speech-to-text software to manually verified transcripts by examining transcription confidence scores, transcription error rates, and machine learning classification accuracy. For the classification tasks, logistic regression, Gaussian naive Bayes, and random forests were used.

**Results:**

The transcription software showed higher confidence scores (*P*<.001) and lower error rates (*P*>.05) for speech from healthy controls compared with patients. Classification models using human-verified transcripts significantly (*P*<.001) outperformed automatically generated transcript models for both spontaneous speech tasks. This comparison showed no difference in the reading task. Manually adding pauses to transcripts had no impact on classification performance. However, manually correcting both spontaneous speech tasks led to significantly higher performances in the machine learning models.

**Conclusions:**

We found that automatically transcribed speech data could be used to distinguish patients with a diagnosis of AD, MCI, or SMC from controls. We recommend a human verification step to improve the performance of automatic transcripts, especially for spontaneous tasks. Moreover, human verification can focus on correcting errors and adding punctuation to transcripts. However, manual addition of pauses is not needed, which can simplify the human verification step to more efficiently process large volumes of speech data.

## Introduction

Identifying individuals with Alzheimer disease (AD) and mild cognitive impairment (MCI) early is beneficial for patient care, family support, and resource planning for the health care system [1]. Identification of individuals who are in the earliest stages of neurodegenerative disease, before irreversible brain changes have occurred, may also allow for the use of disease-modifying therapies when they would be most effective [2].

Analysis of speech to aid in the identification of individuals with early neurodegenerative disease can be a promising strategy, as speech recording is noninvasive, scalable, and easily repeated over time. This contrasts with the current methods for screening for AD or MCI, such as nuclear medicine scans or spinal fluid analysis, which can be both expensive and invasive [3]. Short samples of spontaneous or prompted speech can be collected remotely by telephone or videoconference. To date, speech and language have shown promising results in a significant number of studies aiming to classify AD or MCI [4].

For AD classification using speech, transcription is a key step to leverage the wealth of information contained in lexical data [5,6]. DementiaBank [7], the largest cohort of MCI and AD speech data for research, is entirely manually transcribed. Manual transcription, while highly accurate, is very low throughput (eg, requiring 4 minutes of transcriber time for each minute of audio [8]), limiting the potential scalability and cost savings associated with language-based screening for MCI and AD. As a result, there is a move toward automatically preprocessing medical speech as opposed to manual transcription.

To date, some groups have investigated AD/MCI classification using only automatically generated transcripts produced by transcription software [9,10]. While automatic transcription allows high-throughput speech transcription for a very low cost per sample, these systems can vary in their accuracy (ranging from 68% to 87% in past work [11]), which may affect the performance of downstream linguistic analysis [12]. Furthermore, the impact of automatic preprocessing on classification is not fully understood and should be investigated before continuing downstream investigations.

To better understand the use of automatic transcription for AD/MCI classification, we compared the automatically generated transcripts from Google speech-to-text [13] (“automatic transcripts”) against automatic transcripts that went through a second stage of manual correction (“manually corrected transcripts”). These manually corrected transcripts were used as ground truth. 

Specifically, we first examined a confidence metric in the transcription software for transcribing speech recordings from memory clinic patients versus healthy controls. Second, we measured the word-level accuracy of the automatic transcripts against ground truth. Third, we compared classification performances of machine learning models using data from automatic versus manually-corrected transcripts. Based on these results, we discuss accuracy trade-offs associated with manual transcript verification in the context of dementia classification, and we suggest more efficient manual verification methods to improve the performance of automatically generated transcripts. 

This investigation aims to highlight differences in human versus automatically processed transcripts to drive future automatic transcription–based research. Therefore, we focus here on comparing transcription methods using existing machine learning algorithms rather than building a novel model that outperforms state-of-the-art models.

This work has 4 main contributions addressing knowledge gaps in the existing literature. First, we evaluate automatic transcription and manual transcription on a data set of older adults for AD/MCI classification using 3 measures: transcription confidence, error rates, and machine learning classification accuracy. To our knowledge, this approach for evaluating transcriptions has not been used previously.

Second, our investigation is novel in that we are exploring the robustness of automatic transcription in a cohort of older adults, including those with cognitive impairment and dementia. The aging process includes changes to voice and speech (eg, presbyphonia, word-finding difficulties), which may affect automatic transcription. However, previous investigations on transcription methods have focused solely on younger or heterogeneous cohorts [12,14]. To our knowledge, this is the first investigation on the impact of transcription methods in a cohort of older adults.

Third, based on the evaluation results, we make practical suggestions about how to use automatic transcription. These suggestions will help researchers to better leverage automatic transcription for building natural language processing (NLP)–based screening methods using large data sets for AD/MCI or subjective memory complaints (SMC), which can be a prodromal state for MCI and AD [15].

Finally, while our results are generated with an AD/MCI data set, our findings could also be extrapolated to other neurological and psychiatric conditions where speech analysis is being investigated as a classification tool. This includes stroke [16], Parkinson disease [17], concussion [18], anxiety [19], bipolar disorder [20], depression, and suicidal ideation [21,22].

## Methods

### Overview

**Figure 1:**
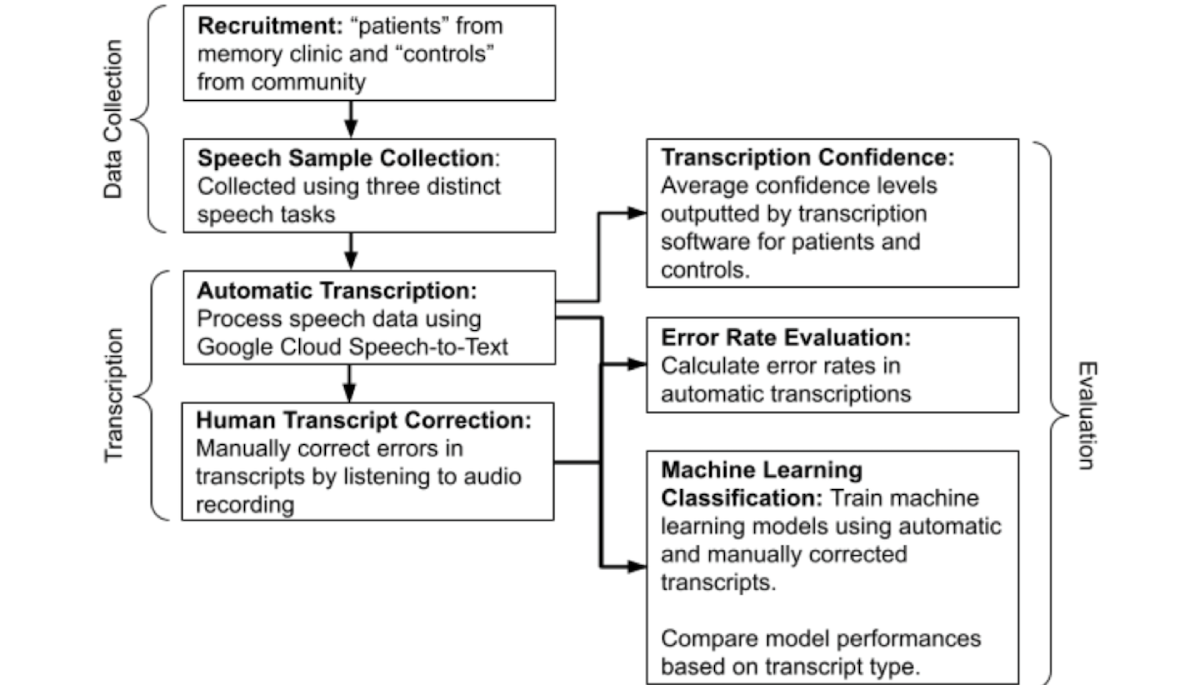
Diagram of our methods and process.

This study involved 3 main phases: (1) data collection, (2) transcription, and (3) evaluation. Our workflow is summarized in Figure 1. As part of a larger study examining machine learning algorithms for classification of memory clinic patients versus healthy controls, we recruited participants with a clinical diagnosis of mild-to-moderate AD, MCI, or SMC (“patients”) from a subspecialty memory clinic and healthy volunteer controls from the community. Participants underwent a test battery that included describing the “Cookie Theft” picture from the Boston Diagnostic Aphasia Examination, a reading task incorporating a sixth-grade level paragraph from the International Reading Speed Texts (IReST), and recounting a pleasant past experience. Their speech was recorded, and we used Google Cloud speech-to-text (STT) to automatically transcribe speech data. We then manually corrected errors in the automatic transcripts. 

For evaluation, we first aggregated transcription confidence levels provided by the software to determine whether transcription software confidence levels vary between patients and controls. Using manually corrected transcripts as the gold standard, we calculated the error rate of automatic transcripts. Then, we compared the performance of machine learning models trained with either automatic or manually corrected transcripts in classifying transcripts as belonging to “patients” versus “controls.”

### Data Collection

#### Recruitment

Patients were recruited from a memory clinic in British Columbia, Canada, and diagnosed with AD, MCI, or SMC. Control participants were recruited from the community, with efforts made to age- and sex-match patient participants. All participants were conversationally fluent in English, could engage in a spontaneous conversation, and were aged 50 or older (mean 68.8, SD 9.5 years). Clinic patients were excluded if they had psychiatric medication changes under 18 months ago or neurological conditions other than SMC, MCI, or AD. We report data from 72 memory clinic patients, of which 44 (30%) were diagnosed with mild-to-moderate AD, 20 (13%) with MCI, and 8 (5%) with SMC (mean age 71.9, SD 8.9 years), along with 77 (52%) healthy volunteers (mean age 65.7, SD 9.1 years). 

Diagnoses were made by specialist clinicians using standard-of-care guidelines. The diagnostic process involves a combination of cognitive testing, neuroimaging, laboratory data, medical history, physical exam, and collateral information collected from individuals close to the patient.

#### Speech Sample Collection

Participants underwent a 10-minute computer-based battery. They were asked to complete a total of 3 speech tasks while their voice was recorded. Participants described the Cookie Theft photo [23], read a standardized paragraph from the IReST, and recalled a pleasant past experience. All tasks were carried out in English. During these spontaneous speech tasks, the audio was recorded using the Logitech C922x ProStream webcam. The Cookie Theft picture description task is a validated spontaneous speech task used extensively in prior work for AD/MCI classification [6,24-26]. This task has also been used for predicting the future risk of developing AD in cognitively normal individuals [27]. 

For the reading task, a single paragraph was selected from IReST, a collection of short paragraphs (<200 words) designed to be readable at a sixth-grade level [28]. To recreate a natural reading environment such as a book or newspaper, the entire paragraph was presented on the screen at the same time rather than displaying each sentence individually, as in some other investigations [29]. For the final task, participants were asked to describe a pleasant past experience (“experience description task”). Several examples were given to participants prior to starting the task, such as their first pet, how they met their best friend, or a place they had traveled.

#### Automatic Transcription

Following the speech tasks, participant audio data was labeled with a unique anonymized identifier and converted to the Waveform audio file format. Next, participant audio was uploaded to the Google Cloud STT platform using US English and 16000 Hz settings, with word-level time stamps enabled, to output the automatic transcripts. 

Each transcribed word was labeled as being within a specific task or as being extraneous from all tasks. Words spoken outside of tasks were removed in downstream experiments. 

#### Human Transcript Correction

After automatic transcript files were generated, human transcribers listened to the recorded audio files and made manual corrections to the transcripts based on the recorded audio. This manual transcription involved 3 steps: fixing transcription errors, adding punctuation, and adding filled pauses and silent pause annotations.  

For the first step, which involved fixing transcription errors**,** human transcribers manually substituted incorrectly transcribed words (eg, change “cookie far” to “cookie jar”), inserted missed words (eg, change “cookie” to “cookie jar”), and deleted extra words (eg, change “cookie key jar” to “cookie jar”). 

The second step entailed adding punctuation. While Google STT adds punctuation, it is very rare, with some transcripts having as few as 0 automatically added punctuation marks. As NLP preprocessing (eg, parsing) benefits from fully formed sentences, human transcribers manually added punctuation (ie, “.”, “!”, and “?”) to the transcripts.

For the third step, which consisted of adding filled pauses and silent pause annotations*,* human transcribers manually added both filled and silent pauses. A filled pause was considered to be any utterance of “uh” or “um.” Filled pauses were consistently transcribed as “uh” or “um” regardless of the length of the pause. Silent pauses were specially labeled as “[pause]” to distinguish this from the word “pause.” Silent pauses were considered to be any break or silence in speech for 0.25 seconds or longer, following Goldman-Eisler [30] and Park [31]. Instances where the participant was not speaking but was not silent were not labeled as a pause (eg, coughing or laughing). The duration of pauses was not differentiated.

**Figure 2:**
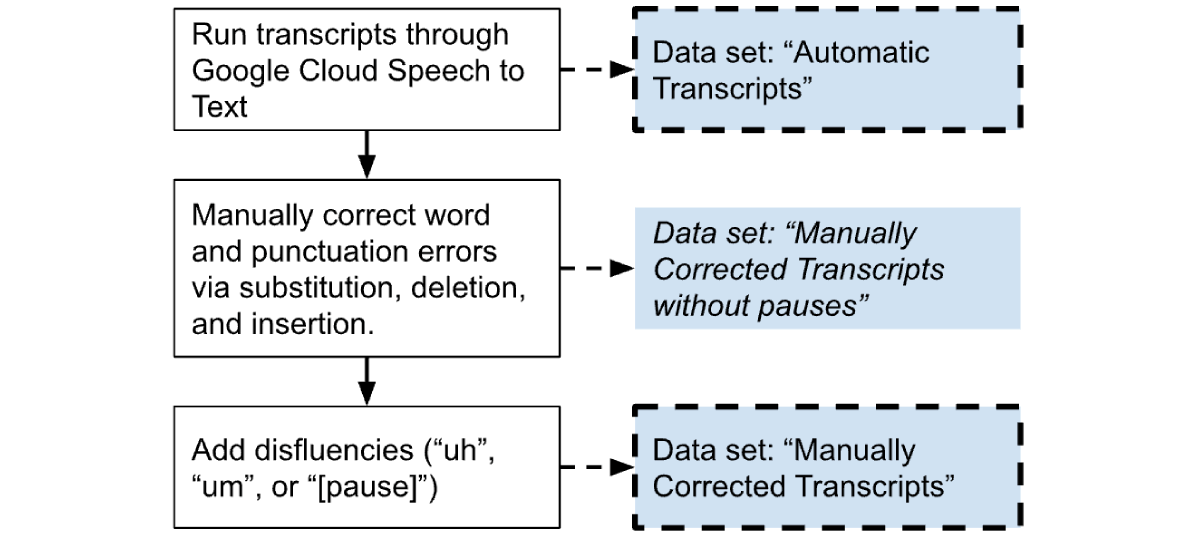
Diagram illustrating how the 3 different transcript data sets were generated.

Figure 2 summarizes the transcription process. Acoustic data were transcribed with Google Cloud STT to generate “automatic transcripts.” Then, human transcribers fixed spoken words and added punctuation based on the audio recording to generate “manually corrected transcripts without pauses.” Finally, human transcribers manually added both filled and silent pauses to generate the “manually corrected transcripts” data set.

### Ethics Approval

This study was approved by the University of British Columbia Clinical Research Ethics Board (H17-02803). All participants provided their written informed consent prior to participating in this study. Baseline demographic characteristics of the patients and controls are summarized in Multimedia Appendix 1.

### Evaluation

#### Transcription Confidence

For a given audio clip, Google STT outputs transcribed words and a confidence level between 0 and 1. This is calculated by aggregating the likelihood values assigned to each word in the audio. A higher number indicates that the words were more likely to be transcribed accurately. We used these confidence levels to determine whether transcription software confidence levels vary between patients and controls and to determine if patient speech was more difficult to transcribe than control speech.

#### Error Rate Evaluation

To examine the error rate of automatic transcripts, we compared these to manually corrected transcripts without pauses. We chose not to include pauses because automatic transcripts do not transcribe pauses at all; thus, not denoting a pause would not be considered an error. 

We calculated standard measures of transcription accuracy, including word error rate (WER) and match error rate (MER) [32], using a Python package, JiWER (v2.1.0, Vassen [33]). These metrics take into account the number of substitutions (eg, “cookie far” to “cookie jar”), deletions (eg, “cookie key jar” to “cookie jar”), and insertions (eg, “cookie” to “cookie jar”) in the manually corrected transcript.

WER represents the rate of errors to the number of input words. This is calculated as follows:



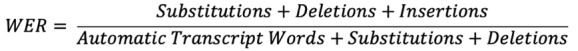



WER does not weigh insertions and deletions equally. For example, a 6-word transcript with 30 insertion errors has a WER value of 5, while a 36-word transcript with 30 deletion errors has a WER of 0.83.

MER represents the probability of a given word match being incorrect and is calculated as follows:



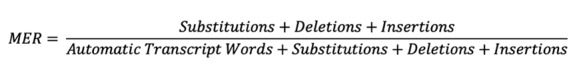



For example, a MER of 0.25 means that 1 out of 4 word matches between the manually corrected transcript and automatic transcript will be an error. MER is calculated similarly to WER. However, MER takes into account the maximum number of words between both the automatic and manually edited transcripts, as opposed to only the number of words in only the automatic transcript. MER also weighs insertions and deletions equally. 

WER and MER were calculated for each individual transcript. Then, the average and standard deviation of these values were calculated for patients and controls and for each task (eg, picture description, reading, and experience description tasks).

#### Machine Learning Classification

To determine whether manual correction impacts machine learning classification of patients versus controls, we performed experiments using both the automatic and manually corrected transcript data sets.

Table 1 outlines the entire feature set by task. For the picture description task and the experience description task, we extracted features from transcripts following the text-based features in previous work [6,34]. These features are based on grammar rules, vocabulary, or psycholinguistics. For the experience description task, we did not include information units used for the picture description task, each of which correspond to visual features in the Cookie Theft picture, such as cookie, jar, boy, or girl. 

**Table 1:** Features for machine learning classification models.

Task	Feature groups and number of features (n) in each group
Picture description	Cookie Theft image information units (13) Part-of-speech (15), context-free-grammar rules (44), syntactic complexity (24), vocabulary richness (4), psycholinguistic (5), repetitiveness (5)
Reading	Syllable count (1), pause count (1)^a^, total duration (1), total time spent speaking (1), proportion of time spent speaking (1), speech rate (1), average syllable duration (1), pauses per syllable (1)^a^, pause rate (1)^a^, pause duration (3)^a^
Experience description	Part-of-speech (15), context-free-grammar rules (44), syntactic complexity (24), vocabulary richness (4), psycholinguistic (5), repetitiveness (5)

^a^These features were computed using acoustic data and transcript data and are also affected by method of pause detection (ie, acoustic vs text data).

For the reading task, we used 12 reading-task–specific features based on the work of Fraser et al [35]. Extracting text features from reading task data may be counterintuitive because each participant reads an identical prompt. However, transcripts may contain repeated words, incorrectly read words, or filled pauses, making transcribed text features potentially informative. Since automatic transcripts do not contain pause information, we first compared automatic transcripts and manually corrected transcripts by using acoustic data to detect unfilled pauses. As an additional comparison for the reading task, we compared using unfilled pauses detected from audio and using unfilled pauses annotated in manually corrected transcripts to determine whether manually adding pauses to transcripts is useful for the reading task or not.

To parse text data and tag parts of speech, we used Stanford CoreNLP [36]. Psycholinguistic features were generated using the MRC database [37], which provides concreteness, familiarity, and imageability scores of English words. Pauses in the reading task were detected using pydub (v0.25.1 [38]), a Python audio processing package. Syllables were detected using Syllables (v1.0.3 [39]), a Python package.

Based on these features, we performed binary classification to distinguish patients from controls. We chose to perform binary classification due to the data size. The number of data samples for finer classes (MCI and SMC) was too small for multiclass classification. To investigate the usefulness of manual correction, we first compared the performance of automatic to manually corrected transcripts. To determine the importance of pause annotation we compared the performance of manually corrected transcripts with and without pauses. 

We tested with 3 classification algorithms that have shown best performances in previous work on dementia classification [40]: logistic regression (LR), random forest (RF), and Gaussian naive Bayes (GNB). In addition, we tested with an end-to-end fine-tuned pretrained model using Bidirectional Encoder Representations from Transformers (BERT) [41] for the picture description and experience description tasks. Note that we did not try BERT models for the reading task because participants read the same text. We used the Python package scikit-learn (v0.19.1 [42]) to perform classification. We used a stratified 10-fold cross-validation approach and repeated this process 10 times in total with differently stratified splits, each generated with a unique random seed. We report the classification performance in terms of area under the receiver operating characteristic curve (AUROC). AUROC is an evaluation metric for classification at various threshold settings and is commonly used for evaluating diagnostic accuracy [43]. The performance metric was averaged over the 10 folds and 10 runs. To remove highly pairwise correlated features and features poorly correlated with the label, we performed correlation feature selection [44]. Highly correlated features were defined as having a Pearson correlation coefficient greater than 0.85, while poorly correlated features had a Pearson correlation coefficient less than 0.20. 

We performed a statistical analysis on the model results to determine if the different transcript data sets led to significant changes in model performance. For each classification algorithm for a given task, we ran a double-sided *t*-test using the null hypothesis that the mean AUROC was no different for automatic and manually corrected transcripts. 

## Results

### Transcription Confidence Results

**Figure 3:**
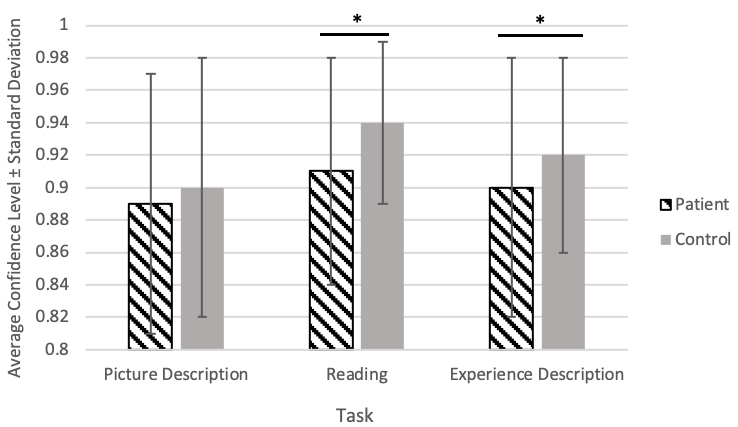
Google speech-to-text confidence results. Error bars represent the standard deviation. * represents *P*<.001, calculated by *t*-test.

Google confidence level results are shown in Figure 3. Generally, Google STT produced a higher confidence level when transcribing audio from controls. In the reading task, for example, the average confidence level was 0.94 (SD 0.05) for controls, compared to 0.91 (SD 0.07) for patients. Both the reading and experience description tasks showed a significantly higher confidence level for controls than patients.

### Error Rate Evaluation Results

**Figure 4:**
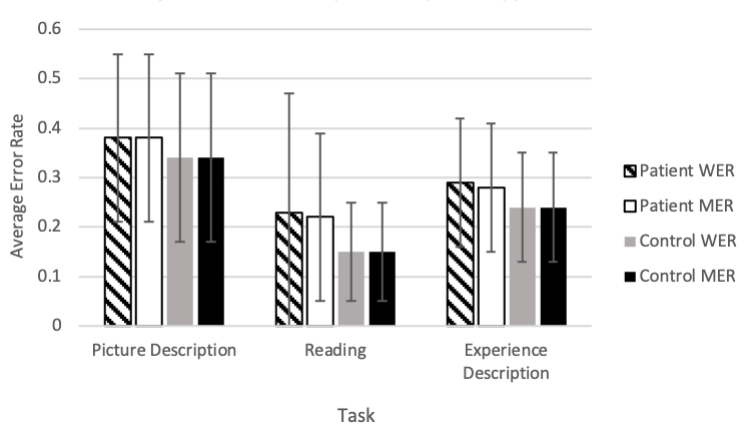
Average error rates by task and participant type. Error bars represent the standard deviation. There were no significant differences in error rates between or within tasks. MER: match error rate; WER: word error rate.

Figure 4 shows the error rate results. In general, automatic transcription had a lower error rate when transcribing control speech compared to patient speech, as shown by the lower average WER and MER. 

The reading task was the most accurate overall, showing an average MER of 0.15 (SD 0.10) for controls and 0.22 (SD 0.19) for patients. This could be because people tend to enunciate when they are asked to read a text aloud. WER and MER results were largely similar overall, suggesting that there were not disproportionately high rates of insertion errors. In other words, manual correction did not involve more word addition as opposed to word deletion or word substitution.

The picture description task was found to have the highest error rate overall when compared to the reading and experience description tasks. This indicates more manual corrections or poorer accuracy of automatic transcription, but it is not clear why this is the case.

### Machine Learning Model Results

Models trained on manually corrected transcripts from the picture description and experience description tasks significantly outperformed models trained on automatic transcripts (Table 2). However, there was no significant difference in model performance trained using either transcription method from the reading task. This finding was true regardless of whether pause-related features were included or not (Table 3).

Table 4 shows results of the models using manually corrected transcripts, with and without pauses for the picture description and experience description tasks. There was no clear trend or significant change in any AUROC result when comparing transcripts with and without pauses. 

**Table 2:** Machine learning classification results of models trained on automatic transcripts compared to results of models trained on manually corrected transcripts.

Task and model type		Automatic transcripts AUROC^a^	Manually corrected transcripts AUROC	Change in AUROC^b^
**Picture description**				
	RF^c^	0.617	0.687	0.070^d^
	GNB^e^	0.662	0.725	0.063^d^
	LR^f^	0.671	0.743	0.072^d^
	BERT^g^	0.618	0.686	0.068^d^
**Experience description**				
	RF	0.503	0.636	0.133^d^
	GNB	0.549	0.677	0.128^d^
	LR	0.543	0.674	0.131^d^
	BERT	0.630	0.650	0.020^d^

^a^AUROC: area under the receiver operating characteristic curve.

^b^Positive change in AUROC indicates that the manually corrected transcript model outperformed the automatic transcript model.

^c^RF: random forest.

^d^Indicates *P*<.001.

^e^GNB: Gaussian naive Bayes.

^f^LR: logistic regression.

^g^BERT: Bidirectional Encoder Representations from Transformers.

**Table 3:** Machine learning classification results of models trained on reading task data with pause features computed using acoustic data or computed using text data.

Reading task	(1) Automatic transcripts AUROC^a,b^	(2) Manually corrected transcripts AUROC^b^	(3) Manually corrected transcripts AUROC^c^	Change in AUROC (3)–(1)
RF^d^	0.638	0.655	0.662	0.024
GNB^e^	0.677	0.677	0.693	0.016
LR^f^	0.589	0.587	0.568	−0.021

^a^AUROC: area under the receiver operating characteristic curve.

^b^Pauses detected from acoustic data.

^c^Pauses detected from text data.

^d^RF: random forest.

^e^GNB: Gaussian naive Bayes.

^f^LR: logistic regression.

**Table 4:** Machine learning classification results of models trained on manually corrected transcripts without pauses compared to results of models trained on manually corrected transcripts (with pauses).

Task and model type		Transcripts without pauses AUROC^a^	Transcripts with pauses AUROC	Change in AUROC^b^
**Picture description**				
	RF^c^	0.666	0.687	0.021
	GNB^d^	0.730	0.725	−0.005
	LR^e^	0.755	0.743	−0.012
	BERT^f^	0.686	0.691	0.005
**Experience description**				
	RF	0.631	0.636	0.005
	GNB	0.676	0.677	0.001
	LR	0.692	0.674	−0.018
	BERT	0.622	0.649	0.027

^a^AUROC: area under the receiver operating characteristic curve.

^b^Positive change in AUROC indicates that the pause model outperformed the no-pause model.

^c^RF: random forest.

^d^GNB: Gaussian naive Bayes.

^e^LR: logistic regression.

^f^BERT: Bidirectional Encoder Representations from Transformers.

## Discussion

### Transcription Confidence

The transcription confidence results showed that the automatic transcription software was consistently more confident in transcribing the speech of controls compared to patients. This may indicate that patient speech differs from the speech used to train the automatic transcription software (which was likely trained using speech from a more general population, including younger or cognitively unimpaired individuals). This may be attributed to the fact that people with Alzheimer disease often have impaired speech production [45], such as distortions (eg, “ook” instead of “cookie”) and phonological paraphasias (eg, “tid” instead of “kid”) [46]. It is especially interesting that the confidence difference between the 2 groups was highest and most significant for the reading task. This confirms that reading task speech is effective for distinguishing AD/MCI patients from controls, as also shown in prior work [35,47,48].

### Error Rate Evaluation

Automatic transcriptions were more accurate for healthy controls compared to patients with AD or MCI, as shown by higher error rate and information loss in patient transcripts. This result is logical in the context of the confidence result, as patient transcripts had significantly lower confidence, meaning that the transcription software was more unsure about its output.

Our results are markedly different from Google’s reports on the error rates of their own software (Google Cloud STT has not disclosed the composition of their training data set). According to Google, their transcription program achieved a WER of 6.7% using 12,500 hours of voice search data and a WER of 4.1% in a dictation task [49]. In contrast, for spontaneous speech tasks, we found a WER range of 24% to 34% for controls and 29% to 38% for patients. The reading task showed a lower WER of 15% for controls and 23% for patients. 

While our error rate results differ from Google’s reported results, they are comparable to the results of other investigations using Google STT derived from simulated medical encounters. Kim et al [50] used audio data from 12 simulated patient and medical student interactions. In this investigation, Google STT showed an average WER of 34%, similar to our WER result of 34% for controls and 38% for patients completing the picture description task. Miner et al [14] recorded audio from 100 patients aged 18-52 (mean age 23) during therapy sessions and found that Google STT had an average WER of 25%. This result is comparable to the WER for our experience description task, which was 29% for patients and 24% for controls. Both therapy-related discussion and the experience description tasks typically involve spontaneous speech with minimal prompting. 

Surprisingly, the experience description task showed lower error rates than the picture description task. This might be because Google STT repeatedly transcribed certain phrases or words related to the picture incorrectly across participants, leading to a higher average error rate in this task. It is also possible that the experience description is easier for automatic transcription because it is more conversational, like the material Google STT may have been trained on. Further investigation into to the discrepancy in performance between different spontaneous speech tasks is warranted.

The reading task WER for our cohort was notably higher than previous research. Kepuska et al [51] used Google STT to transcribe audio from 630 speakers reading 10 sentences each and found an average WER of 9%. This is markedly lower than the results of our investigation, in which we found that the 9-sentence reading task produced a WER of 23% for patients and 15% for controls. One possible reason for this large disparity is that Google STT is not specific to a particular population (eg, older adults may experience normal age-related changes to the larynx and vocal cords over time, known as presbyphonia) and may produce more accurate transcriptions in a more generalized sample. 

### Machine Learning Models

Despite our previous results showing that automatic transcription for our data set is more inaccurate than values reported by Google, our machine learning model results still show that automatic transcripts are discriminative for AD/MCI. Other studies using automatic transcription for classification experiments have noted that inaccuracies or errors in the audio-to-text transcription do not necessarily affect classification results [52]. 

However, manually correcting the picture description and experience description task transcriptions led to significantly higher performances in the machine learning models. By comparison, both automatic and manually corrected reading task transcripts showed similar performance, likely due to the majority of reading features being computed from audio data. To address this concern, we examined text versus audio-based silent pause detection and again found no significant changes in performance. This indicates that using either audio or text to detect pauses will produce similar results and that manually correcting transcripts does not significantly change model performance.

Surprisingly, the addition of filled and silent pauses did not significantly change performance for any of the tasks and algorithms. Moreover, using the pauses from the transcripts showed similar classification results to using pauses detected from audio data for the reading task. Previous studies have shown that people with Alzheimer disease demonstrate a multitude of disfluencies in their speech, including pauses [53-55]. However, manually adding pauses as either words (“um” or “uh”) or tokens (“[pause]”) to transcripts did not seem to have any effect on classification models. This could be because older adults also experience age-related changes in their speech, such as an increase in silent pauses [56], potentially weakening the association of pauses to either the patient or control category. Alternatively, this result may be due to the fact that there are no features that “directly” model pauses for the description tasks, weakening the association of the tasks with pauses. 

### Limitations

Some limitations with our cohort include varying language ability and variations between transcribers. In our cohort, English was not the first language of 13% of the patients and 21% of the control group, which could potentially contribute to transcription errors. Additionally, our use of 3 different transcriptionists may have introduced interrater variability, especially for more subjective correction steps such as adding punctuation, although variation in manual transcription was controlled via inter-transcriptionist review and protocol development for standardized transcription. Another limitation of our investigation is the size of the data set (N=149), which is quite small for machine learning experiments. However, this is an issue facing most work on using machine learning for dementia classification, especially with newly built data sets (N=55-82) [5,29,35]. While the DementiaBank and ADReSS data sets are larger (N=287 with 687 samples and N=156, respectively), they were originally created in the mid-1980s and are limited by the diagnostic practices of that time. The work described herein aims to mitigate this challenge. Our best practice suggestions for automatic transcription will facilitate data collection at a much faster rate in the future.

It is also important to note that this investigation was completed using Google speech-to-text software in an English-speaking cohort. Competitor speech-to-text software may produce different results, so readers should be wary when applying our conclusions to other software. Applying a similar method to a non-English data set may also produce different results, especially because automatic transcription in other languages might not be as advanced as English. Finally, speech-to-text software is continually being refined and improved. In the future, automatically generated transcripts may be indistinguishable from human-generated transcripts. In the meantime, it is still valuable to understand the impacts of automatic transcription, especially for medical speech data sets.

### Conclusion

Our results showed that automatically transcribed speech data from a web-based speech recognition platform can be effectively used to distinguish patients from controls. According to our results, to improve the classification performance of automatically generated transcripts, especially those generated from spontaneous speech tasks, a human verification step is recommended. Our analyses indicate that human verification should focus on correcting errors and adding punctuation to transcripts and that manual addition of pauses is not needed, which can simplify the human verification step to more efficiently process large volumes of speech data. 

## Appendix

Multimedia Appendix 1 Summary baseline characteristics of patients and controls.

## Abbreviations

AD: Alzheimer disease
AUROC: area under the receiver operating characteristic curve
BERT: Bidirectional Encoder Representations from Transformers
GNB: Gaussian naive Bayes
IReST: International Reading Speed Texts
LR: logistic regression
MCI: mild cognitive impairment
MER: match error rate
NLP: natural language processing
RF: random forest
SMC: subjective memory complaints
STT: speech-to-text
WER: word error rate
