# Supplementary material for: Evaluating Web-Based Automatic Transcription for Alzheimer Speech Data: Transcript Comparison and Machine Learning Analysis
Source: JMIR Aging. 2022 Sep 21;5(3):e33460. doi: 10.2196/33460 (PMC9536526; doi:10.2196/33460)
Supplement: Multimedia Appendix 1 [file aging_v5i3e33460_app1.docx]

|  | Patients (n=72) | Controls (n=77) |
| --- | --- | --- |
|  |  |  |
| Mean age (± standard deviation) | 71.9 ± 8.9 | 65.7 ± 9.1 |
| Age range | 53-96 | 50-92 |
| Number of females (% female) | 34 (47%) | 55 (71%) |
| English as a first language | 63 | 61 |
| Mean MoCA Score (± standard deviation) | 20.7 ± 5.6 | 27.2 ± 2.5 |
| MoCA Score Range | 5-30 | 19-30 |
